# Intermittent Ophthalmia

**Published:** 1830-04-01

**Authors:** 


### XLIII.

### Intermittent Ophthalmia.

Periodical *pains* are generally soon detected as such, and the patient is spared much effusion of blood; but when symptoms of inflammation accompany the neuralgic affection, the periodicity of the complaint is too often overlooked, or disregarded, and depletory measures are carried to an extent that increases the evil, and protracts its cure. The following case is deserving of record.

A man turned 30 years of age, had been a soldier, but, for some years prior to 1827, had worked in a cotton manufactory. In the Spring of the last mentioned year he became affected with a severe ophthalmia, first of the right, and subsequently of the left eye. This at length subsided; but in three months afterwards, the inflammation re-appeared in the right eye, with a periodicity of eight days:—in the following manner. After being some hours in bed, the patient was awoke by violent pain in the eye, accompanied by lachrymation, redness, and such a sense of distention that he could scarcely be persuaded that the eye was not bursting from the socket. The feeling of sand in the eye was also very distressing. These symptoms would continue during the succeeding day, till towards the evening, when the pains would diminish and ultimately cease, leaving the eye in a state of complete epiphora. On the third day the organ would appear quite sound. Some degree of aversion to light, and lachrymation, however continued occasionally, during the intermissions, especially in particular states of the weather. These paroxysms returned every eighth day. In the Winter of 1827, the accessions began to come on in the afternoon, instead of the middle of the night, continuing with great severity till the next morning, and preventing sleep. In the intervals, he carried on his usual labours. On the 8th of April, 1828, he received a blow on the left eye, which instantly deprived him of sight. He did not apply for medical assistance till the 19th of May, when marks of inflammation were visible both in the conjunctiva and sclerotica, with an ulceration on the transparent cornea. The anterior chamber of the eye was very turgid with aqueous humours—the eye prominent, and the pupil drawn obliquely downwards and outwards, while it was remarkably contracted. At this time, he said nothing about the preceding periodical affection of the right eye, and the case was treated as one of traumatic ophthalmia. But the old intermitting affection of the right eye now returned, and with intervals of three instead of eight days. The periodicity at length was so remarkable that proper means were used, and the disease stopped.*

Such cases are more common than are imagined. Within these few weeks we were requested to visit a poor artisan who, in the year 1829, was laid up with a complaint of the above kind for seven or eight weeks, to the almost ruin of his family. The pain was on the left side of the head, and the left eye became every day red and apparently inflamed, with excessive lachrymation and intolerance of light. Repeated depletion and the usual antiphlogistic means did no good, but rather aggravated the complaint, which appeared to have given way to time rather than physic. When we first saw this patient, in the height of a paroxysm, such was the apparent intensity of inflammation in the eye, that we ordered a repetition of leeches to the temple. We soon found out our mistake, and ascertained that the malady came on regularly every morning at six o'clock. The arsenical solution and quinine cut short the complaint in three days, and the man returned to his work, rejoicing that this year's attack was so short, in comparison with that of the preceding year. Many such cases have come under our notice during the last three or four years, and we are convinced that no one would suspect the real nature of the complaint unless he happened to notice the periodicity. The phenomena are exactly those of inflammation, excepting that the pain is more excessive than in common ophthalmia. Yet we really believe that the term inflammation here, is hardly warrantable. It is rather an afflux of blood to the part, occasioned by, and no doubt augmenting the intensity of the nervous pain—the vascular phenomena rapidly subsiding when the neuralgic orgasm is over. The effects of genuine inflammation would not—could not, so suddenly disappear.

* Journal Complementaire, Jan. 1830.

